# Cisplatin/gemcitabine or oxaliplatin/gemcitabine in the treatment of advanced biliary tract cancer: a systematic review

**DOI:** 10.1002/cam4.299

**Published:** 2014-08-11

**Authors:** Frédéric Fiteni, Thierry Nguyen, Dewi Vernerey, Marie-Justine Paillard, Stefano Kim, Martin Demarchi, Francine Fein, Christophe Borg, Franck Bonnetain, Xavier Pivot

**Affiliations:** 1 Department of Medical Oncology, University Hospital of BesançonBesançon, France; 2 Methodology and Quality of Life in Oncology Unit, University Hospital of BesançonBesançon, France; 3 Department of Medical Oncology, Hospital of MontbéliardMontbéliard, France; 4 Department of Gastroenterology, University Hospital of BesançonBesançon, France; 5 INSERM, Unit 1098, University of Franche-ComtéBesançon, France; 6 EA 3181 University of Franche-ComtéBesançon, France

**Keywords:** Biliary tract cancer, cisplatin, gemcitabine, oxaliplatin

## Abstract

Cisplatin/gemcitabine association has been a standard of care for first-line regimen in advanced biliary tract cancer nevertheless oxaliplatin/gemcitabine regimen is frequently preferred. Because comparative effectiveness in clinical outcomes of cisplatin- versus oxaliplatin-containing chemotherapy is not available, a systematic review of studies assessing cisplatin/gemcitabine or oxaliplatin/gemcitabine chemotherapies in advanced biliary tract cancer was performed. Published studies evaluating cisplatin/gemcitabine or oxaliplatin/gemcitabine in advanced biliary tract cancer were included. Each study was weighted according to the number of patients included. The primary objective was to assess weighted median of medians overall survival (mOS) reported for both regimens. Secondary goals were to assess weighted median of medians progression-free survival (mPFS) and toxic effects were pooled and compared within each arm. Thirty-three studies involving 1470 patients were analyzed. In total, 771 and 699 patients were treated by cisplatin/gemcitabine and oxaliplatin/gemcitabine, respectively. Weighted median of mOS was 9.7 months in cisplatin group and 9.5 months in oxaliplatin group. Cisplatin-based chemotherapy was significantly associated with more grade 3 and 4 asthenia, diarrhea, liver toxicity, and hematological toxicity. Sensitivity analysis including only the studies with the standard regimen of cisplatin (25–35 mg/m^2^ administered on days 1 and 8) showed that the weighted median of mOS increased from 9.7 to 11.7 months but Gem/CDDP regimen remained more toxic than Gemox regimen. These results suggest that the Gem/CDDP regimen with cisplatin (25–35 mg/m^2^) administered on days 1 and 8 is associated with survival advantage than Gemox regimen but with addition of toxicity.

## Introduction

Biliary tract carcinomas (BTCs) are invasive adenocarcinomas that arise from the epithelial cells of the biliary tree, which comprises intrahepatic and extrahepatic bile ducts, and gallbladder. Even though BTCs are considered as rare tumors, they represent about 30% of the total primary liver cancers with an incidence rate close to that of hepatocarcinoma. Approximately 1200 new cases in the United Kingdom and 9000 in the United States are diagnosed per year [1]. Unfortunately, only a minority of patients diagnosed with these aggressive tumors present at an early resectable stage, and disease recurrence rates are high despite curative-intent surgery. Prognosis of patients with advanced BTC is extremely poor with overall survival (OS) less than 1 year.

Chemotherapy is a palliative treatment option for patients with advanced disease. Different drugs has demonstrated activity in BTC, including fluoropyrimidines, gemcitabine, cisplatin, and oxaliplatin [2–6]. A pooled analysis from Eckel et al. [7] including 104 trials with 2810 patients, has established gemcitabine combined with platinum compounds as the provisional standard of chemotherapy in advanced biliary tract cancer.

In 2010, a randomized multicentric phase III ABC-02 trial established the cisplatin/gemcitabine (Gem/CDDP) combination as standard chemotherapy regimen in advanced BTC [8]. The OS was 11.7 months compared to 8.1 months in gemcitabine single agent arm (HR, 0.64; 95% CI, 0.52–0.80; *P* < 0.001). Different oxaliplatin/gemcitabine (Gemox) combination regimens were assessed in several phase II clinical trials. One randomized study evaluated efficacy of modified gemcitabine and oxaliplatin (mGEMOX) regimen versus best supportive care or fluorouracil and folinic acid (FUFA) regimen. Median OS was 9.5, 4.5, and 4.6 months for mGEMOX, BSC, and FUFA (*P* = 0.039), respectively [9].

Since the ABC-02 trial, Gem/CDDP regimen has become a standard of care in first-line treatment. However, Gemox regimen is a well-established regimen since Sharma's study. Furthermore, several clinical randomized trials use Gemox as the comparative arm [10, 11]. These two regimens have never been compared. In this context, we carried out this systematic review to obtain an overall descriptive view of efficacy and safety of Gem/CDDP and GEMOX regimens in the first-line chemotherapy treatment of advanced BTC.

## Methods

### Search for trials

Literature searches in PubMed, American Society of Clinical Oncology (ASCO), and European Society of Medical Oncology databases were performed. Searches were limited to human studies and English-language publications. For PubMed database research, the following strategies were used: (“cholangiocarcinoma” OR “biliary tract carcinoma” OR “biliary tract cancer” OR “gall bladder cancer” OR “gall bladder carcinoma”) AND (((gemcitabine) AND oxaliplatin) OR ((gemcitabine) AND “cisplatin”)). The main keywords used for the search on ASCO and ESMO database are cholangiocarcinoma and chemotherapy.

### Selection criteria

Eligible trials included patients with locally advanced or metastatic biliary tract cancers, defined as tumors of the gallbladder and intrahepatic, perihilar, distal bile ducts, and ampullary tumors. Studies assessed first-line chemotherapy by Gem/CDDP or Gemox.

### Data extraction

Two authors (F. F., M. J. P.) independently extracted information using predefined data abstraction forms. The following details were extracted: type of study, year of publication, study period, number of centers, nationality of the centers, follow-up, eligibility criteria, doses of chemotherapy, treatment schedule, duration of the treatment, patients' characteristics (age, sex, extent of disease, primary tumor site, WHO-PS, metastatic sites), primary endpoint and its definition, secondary endpoints and their definitions, overall survival (definition, median, and 95% confidence interval), progression-free survival (definition, median, and 95% confidence interval), and grade 3 and 4 toxicity data.

Missing data from studies deemed potentially eligible were sought from the authors via e-mail request. All data were checked for internal consistency, and disagreements were resolved by discussion among the investigators.

### Statistical analysis

Quantitative data were compared using a Student's test or a Mann and Whitney's test as appropriate. Qualitative data were compared using chi-square test or Fisher's exact test.

Patients characteristics (age, sex, extent of disease, primary tumor site, WHO-PS >2, metastatic locations) were pooled and compared within each arm.

The primary objective was to assess median of medians overall survival (mOS) and a weighted mOS in studies evaluating Gemox regimens and Gemcitabine/CDDP regimens. The secondary objectives were to assess median of medians progression-free survival (mPFS) and a weighted mPFS, to pool and compare adverse events within each arm. The weighted approach, based on the number of patients included, took into consideration the study size. Thus, larger study contributed more than smaller studies. Toxic effects according to the National Cancer Institute's Common Toxicity Criteria for Adverse Events grade 3 and 4 were pooled and compared within each arm.

In order to assess the internal validity of our results, these analyses were repeated 1000 times with the use of bootstrap sample to derive 95% confidence interval for the mOS and mPFS in Gemox and Gemcitabine/CDDP groups.

*P*-value of 0.05 or lower was considered as statistically significant. Analyses were conducted with the use of SAS software, version 9.2 (SAS Institute Inc., Cary, NC), and R software (version 2.10.1).

## Results

### Characteristics of the studies

**Figure 1:**
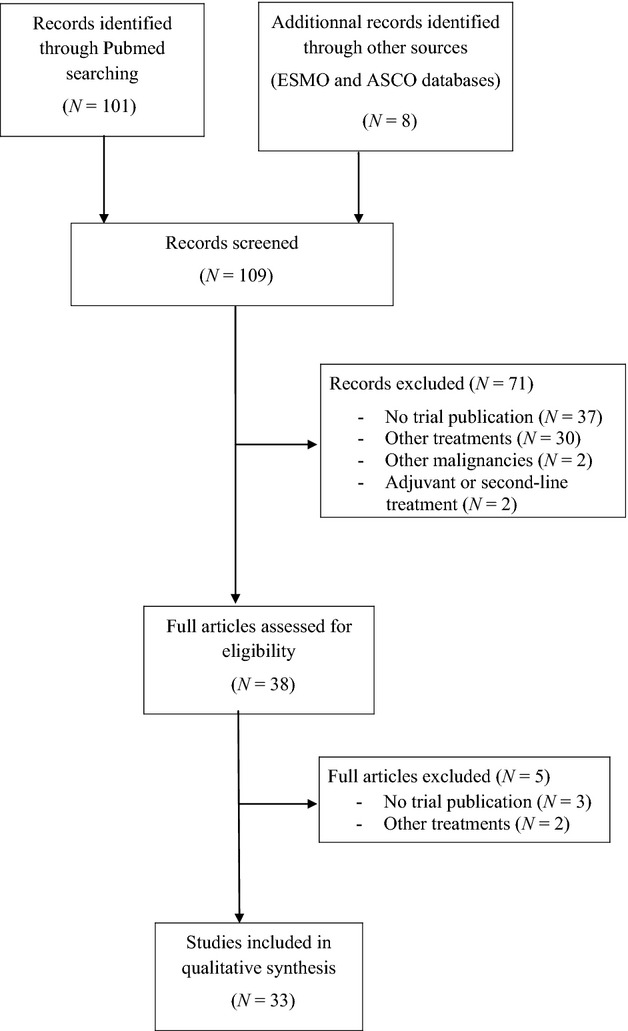
Flowchart showing the progress of trials through the review.

Thirty-three studies were included in the review (Fig. 1). Baseline characteristics of the 33 studies are listed in Tables 1 and 2. They were published between 2001 and September 2012. Gem/CDDP and Gemox regimens were investigated in 18 and 15 studies, respectively. Among the 18 studies evaluating Gemcitabine/CDDP, two studies were retrospective analyses, 13 studies were single arm phase II trials, two studies were randomized comparative phase II trials, and one study was a phase III trial. Among the 15 studies assessing Gemox, three studies were retrospective analyses, eight studies were single arm phase II trials, three studies were a randomized comparative phase II trials, and one study was a phase III trial. In total, 771 and 699 patients were treated by Gem/CDDP and Gemox, respectively. Table 3 pools patients' characteristics by arms. The only significant difference among available data was the stage of disease: 73% versus 57% metastatic patients in Gem/CDDP and Gemox groups, respectively (*P* < 0.0001).

**Table 1:** Characteristics of 18 studies assessing combination of cisplatin/gemcitabine.

First author	Type of study	Overall patient number	Year of publication	Patients number by arm	Chemotherapy regimen	Dose (mg/m²)	Treatment schedule	Treatment duration
Carraro [12]	Phase II	11	2001	11	Cisp + Gem	30 + 1000	d1d8d15/d1d8d15-q4w	NA
Malik [13]	Phase II	11	2003	11	Cisp + Gem	70 + 1000	d1/d1d8-q3w	Until DP or UT
Baluch [14]	Phase II	14	2003	14	Cisp + Gem	60 + 1000	d1/d1d8-q3w	NA
Reyes-Vidal [15]	Phase II	44	2003	44	Cisp + Gem	35 + 1250	d1d8/d1d8-q3w	NA
Doval [16]	Phase II	30	2004	30	Cisp + Gem	70 + 1000	d1/d1d8-q3w	Six cycles unless DP or UT
Thongprasert [17]	Phase II	43	2005	43	Cisp + Gem	75 + 1250	d1/d1d8-q3w	Until DP or UT
Kim [18]	Phase II	29	2006	29	Cisp + Gem	60 + 1250	d1/d1d8-q3w	Until DP
Giuliani [19]	Phase II	38	2006	38	Cisp + Gem	80 + 1000	d1/d1d8-q3w	Six cycles unless DP or UT
Park [20]	Phase II	27	2006	27	Cisp + Gem	75 + 1000	d1/d1d8d15-q4w	NA
Lee [21]	Phase II	24	2006	24	Cisp + Gem	70 + 1000	d1/d1d8-q3w	Until DP or UT
Meyerhardt [22]	Phase II	33	2007	33	Cisp + Gem	30 + 1000	d1d8/d1d8-q3w	Until DP or UT
Charoentum [23]	Retrospective study	42	2007	42	Cisp + Gem	75 + 1250	d1/d1d8-q3w	NA
Lee [21]	Phase II	35	2008	35	Cisp + Gem	70 + 1250	d1/d1d8-q3w	Eight cycles
Valle [24]	Randomized comparative Phase II	86	2009	42	Cisp + Gem	25 + 1000	d1d8/d1d8-q3w	Eight cycles unless DP or UT
				44	Gem	1000	d1d8d15-q4w	Six cycles unless DP or UT
Goldstein [25]	Phase II	50	2010	50	Cisp + Gem	20 + 1000	d1d8/d1d8-q3w	Until DP or UT
Okusaka [26]	Randomized comparative Phase II	83	2010	41	Cisp + Gem	25 + 1000	d1d8/d1d8-q3w	Sixteen cycles unless DP or UT
				42	Gem	1000	d1d8d15-q4w	Twelve cycles unless DP or UT
Valle [8]	Phase III	410	2010	204	Cisp + Gem	25 + 1000	d1d8/d1d8-q3w	Eight cycles unless DP or UT
				206	Gem	1000	d1d8d15-q3w	Six cycles unless DP or UT
Weatherly [27]	Retrospective study	85	2011	53	Cisp + Gem	NA	NA	NA
				32	“Alternative” regimens	NA	NA	NA

DP, disease progression; UT, unacceptable toxicity.

**Table 2:** Characteristics of 15 studies assessing combination of oxaliplatin/gemcitabine.

First author	Type of study	Overall patient number	Year of publication	Patients number by arm	Chemotherapy regimen	Dose (mg/m²)	Treatment schedule	Treatment duration
Gebbia [28]	Phase II	24	2005	24	Ox + Gem	100 + 1000	d1/d1d8-q3w	NA
Harder [29]	Phase II	31	2006	35	Ox + Gem	100 + 1000	d1d15/d1d8d15-q4w	Until DP or UT
Verderame [30]	Phase II	24	2006	24	Ox + Gem	100 + 1000	d1/d1d8-q3w	Until DP or UT
Manzione [31]	Phase II	34	2007	34	Ox + Gem	100 + 1000	d2/d1-q2w	Until DP or UT
Kim [32]	Phase II	40	2008	40	Ox + Gem	100 + 1000	d2/d1-q2w	Until DP or UT
Andre [33]	Phase II	70	2008	70	Ox + Gem	100 + 1000	d2/1-q2w	Until DP or UT
Cassier [34]	Retrospective study	76	2008	39	Ox + Gem	NA	NA	NA
				26	Gem	NA	NA	NA
				11	FU	NA	NA	NA
Jang [35]	Phase II	53	2010	53	Ox + Gem	100 + 1000	d1/d1d8-q3w	Until DP or UT
Hollebecque [36]	Retrospective study	44	2010	44	Ox + Gem	100 + 1000	d2/d1-q2w	Until DP or UT
Sharma [37]	Phase II	48	2010	48	Ox + Gem	80 + 900	d1d8/d1d8-q3w	Six cycles unless DP or UT
Sharma [9]	Randomized comparative Phase II	82	2010	26	Ox + Gem	80 + 900	d1d8/d1d8-q3w	Six cycles unless DP or UT
				27	BSC			
				28	FU + FA	425 + 20	d1-q1w	Six cycles unless DP or UT
Lee [32]	Phase III	268	2011	133	Ox + Gem	100 + 1000	d2/d1-q2w	Until DP or UT
				135	Ox + Gem + erlotinib	100 + 1000 + 100 mg	d2/d1-q2w/daily	Until DP or UT
Fiteni [38]	Retrospective study	44	2011	44	Ox + gem	100 + 1000	d1/d1-q2w	Until DP or UT
Phelip [39]	Randomized comparative Phase II	34	2012	16	Ox + Gem	100 + 1000	d1/d1-q2w	6 months
				18	RT-CT			Until the end of RT
Malka [40]	Randomized comparative Phase II	150	2012	74	Ox + Gem	100 + 1000	d2/d1-q2w	NA
				76	Ox + Gem + Cetuximab	100 + 1000 + 500	d2/d1/d1-q2w	NA

DP, disease progression; UT, unacceptable toxicity.

**Table 3:** Patient characteristics according to treatment arm.

	Cisplatin/gemcitabine (*N* = 771)			Oxaliplatin/gemcitabine (*N* = 699)			*P*
		
	No. of missing studies	No. of available data		No. of missing studies	No. of available data		
Age-(median in years)	4		58.15 ± 5.2	4		61.1 ± 5.6	0.1693
Male sex, *n* (%)	3	660	353 (53)	3	571	271 (47)	0.03958
Disease stage	9	437		8	259		
Locally advanced			118 (27)			111 (43)	
Metastatic			319 (73)			148 (57)	<0.0001
Primary tumor site, *n* (%)	7	539		5	496		
Voie biliaire			341 (63)			327 (66)	
Vésicuel biliaire			179 (33)			160 (32)	
Ampoule de vater			19 (4)			9 (2)	0.2108
WHO-PS-n >2 (%)	6	545	4 (0)	6	466	0 (0)	0.1288
Metastatic sites, *n* (%)							
Peritoneal carcinomatosis	14	151	21 (14)	12	226	27 (12)	0.6369
Intraperitoneal	12	236	108 (46)	11	261	133 (51)	0.2809
Liver	13	193	135 (70)	10	283	196 (69)	0.9193
Lung	13	186	25 (13)	10	301	43 (14)	0.8931

### Overall survival

**Figure 2:**
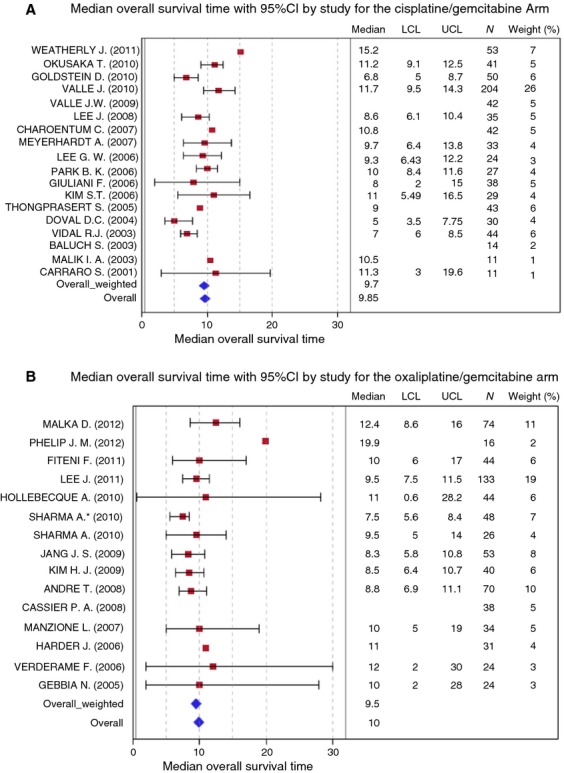
Overall survival with (A) cisplatin/gemcitabine and (B) oxaliplatin/gemcitabine.

Data on OS were available for 16 studies in Gem/CDDP group and 14 in Gemox group. Individual medians OS and their confidence intervals were plotted for each study within the two groups (Fig. 2A and B).

Median of mOS was 9.85 months (ranges: 5–15.2 months) (bootstrap interquartile 95% CI: [8.6–11]) in Gemcitabine/CDDP group and 10 months (ranges: 7.5–12.4 months) (bootstrap interquartile 95% CI: [8.8–11]) in Gemox group.

Weighted median of mOS was 9.7 months in Gem/CDDP group (bootstrap interquartile 95% CI: [9–10.5]) and 9.5 months (bootstrap interquartile 95% CI: [9.5–10]) in Gemox group.

### Progression-free survival

**Figure 3:**
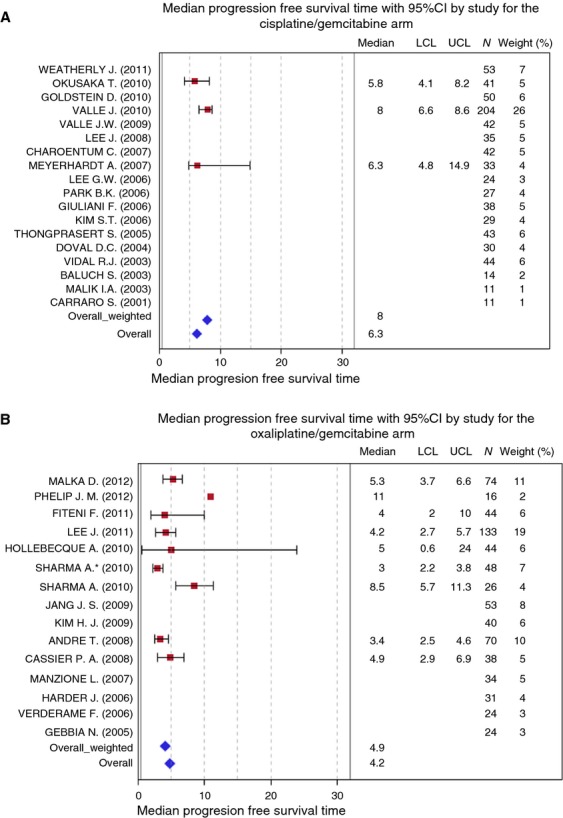
Progression-free survival with (A) cisplatin/gemcitabine and (B) oxaliplatin/gemcitabine.

Data on PFS were available for three studies in Gem/CDDP group and nine in Gemox group. Individual mPFS and their confidence intervals were plotted for each study within the two groups (Fig. 3A and B).

Median of mPFS was 6.3 months (range: 4–8.5 months) (bootstrap interquartile 95% CI: [5.8–8]) in Gemcitabine/CDDP group and 4.9 months (range: 3.4–5.3 months) (bootstrap interquartile 95% CI: [3.5–8.5]) in Gemox group.

Weighted median of mPFS was 8 months in Gem/CDDP group (bootstrap interquartile 95% CI: [8–8]) and 4.2 months in Gemox group (bootstrap interquartile 95% CI: [4.2–4.9]).

Individual data for each study are presented in Figure 3A and B.

### Toxicity

The number of NCI-CTC grade 3 and 4 adverse events was pooled by arms. Patients treated with Gemcitabine/CDDP compared with patients treated with Gemox were more likely to experience asthenia (16% vs. 6%, *P* < 0.0001), diarrhea (6% vs. 2%, *P* = 0.02919), hepatotoxicity (11% vs. 7%, *P* = 0.04761), anemia (15% vs. 6%, *P* < 0.0001), thrombopenia (17% vs. 7%, *P* < 0.0001), and neutropenia (34% vs. 12%, *P* < 0.0001). On the other hand, oxaliplatin-based chemotherapy caused more peripheral neuropathy than that gemcitabine/CDDP regimens (11% vs. 0%, *P* < 0.0001) (Table 4).

**Table 4:** Adverse events (grade 3 and 4) according to treatment groups.

	Cisplatin/gemcitabine (*N* = 771)			Oxaliplatin/gemcitabine (*N* = 699)			*P*
		
	No. of missing studies	No. of available data	*N* (%)	No. of missing studies	No. of available data	*N* (%)	
Asthenia	12	381	61 (16)	8	408	23 (6)	<0.0001
Nausea	9	500	23 (5)	10	331	12 (4)	0.5976
Vomiting	8	538	26 (5)	6	519	18 (3)	0.2845
Diarrhea	10	266	15 (6)	8	373	8 (2)	0.02919
Peripheral neuropathy	13	176	1 (0)	3	543	58 (11)	<0.0001
Alopecia	14	295	5 (2)	13	97	0 (0)	0.3388
Renal toxicity	10	424	7 (2)	13	97	0 (0)	0.358
Hepatotoxicity	10	451	49 (11)	9	360	24 (7)	0.04761
Anemia	5	608	94 (15)	8	300	17 (6)	<0.0001
Thrombopenia	3	661	110 (17)	6	473	34 (7)	<0.0001
Neutropenia	4	634	216 (34)	7	442	52 (12)	<0.0001
Mucositis	13	165	3 (2)	11	241	0 (0)	0.0664
Febrile neutropenia	12	200	8 (4)	10	331	13 (4)	1

### Sensitivity analysis

While the oxaliplatin-based regimens are relatively homogeneous (oxaliplatin dose range 80–100 mg/m^2^), there is marked heterogeneity of the cisplatin–gemcitabine regimens included in the review with variance of the cisplatin doses from low-dose (25–35 mg/m^2^) to high-dose regimens (60–80 mg/m^2^). Therefore, in the sensitivity analysis we assessed the OS, PFS, and toxicity of the Gem/CDDP group by including only the six studies with cisplatin low-dose (25–35 mg/m^2^) administered on days 1 and 8 [8, 14, 22, 24–26] as in the pivotal phase III ABC-02 trial.

Weighted median of mOS increased from 9.7 to 11.7 months (bootstrap interquartile 95% CI: [11.2–11.7]). Weighted median of mPFS was similar to the previous analysis; 8 months in Gem/CDDP group (bootstrap interquartile 95% CI: [8–8]).

Patients treated with Gemcitabine/CDDP compared with patients treated with Gemox remained more likely to experience asthenia (16% vs. 6%, *P* < 0.0001), diarrhea (8% vs. 2%, *P* = 0.004), hepatotoxicity (15% vs. 7%, *P* = 0.0006), anemia (13% vs. 6%, *P* = 0.002), thrombopenia (14% vs. 7%, *P* = 0.043), and neutropenia (29% vs. 12%, *P* < 0.0001). Peripheral neuropathy rate remained more important in the oxaliplatin-based chemotherapy group (11% vs. 1%, *P* = 0.002) (Table S1).

## Discussion

Since the randomized multicentric phase III ABC-02 trial, Gem/CDDP combination is considered as the standard first-line chemotherapy in advanced BTC [8]. However, Gemox chemotherapy is frequently preferred as first-line chemotherapy in many cancer institutions and is frequently used in recent clinical trials in association with biotherapies in exploratory studies and as the comparative arms in randomized [10, 11]. The reason of choice is based on the easier administration of oxaliplatin than cisplatin requiring hyperhydration and expose to higher risk of renal toxicity. Nevertheless, superiority of one platinum compound over another in this setting was not demonstrated, and there is no clinical trial with direct comparison between different platinum salts in advanced BTC.

This review was conducted following the PRISMA guidelines. It was an exhaustive review including 33 studies (involving 1470 patients) which assessed Gem/CDDP regimen (18 studies involving 771 patients) or Gemox regimen (15 studies involving 699 patients) for advanced BTC. In clinical practice, Gemox and Gem/CDDP are frequently used as first-line therapy in advanced BTC but they have never been compared. A direct statistical comparison was not feasible but to obtain an overall view of comparing efficacy and safety between Gem/CDDP and Gemox regimens, we conducted a descriptive statistical approach by assessing the weighted median of mOS and the weighted median of mPFS. Individual results from each study were presented in forest plots. Weighted median of mOS was 9.7 months in Gem/CDDP group and 9.5 months in Gemox group.

Nevertheless, the oxaliplatin-based regimens are relatively homogeneous (oxaliplatin dose range 80–100 mg/m^2^), but there is marked heterogeneity of the cisplatin–gemcitabine regimens included in the review with variance of the cisplatin doses from low-dose (25–35 mg/m^2^) to high-dose regimens (60–80 mg/m^2^). Therefore, we performed a sensitivity analysis by including only the studies with standard regimen of cisplatin (25–35 mg/m^2^ administered on days 1 and 8) as in the pivotal phase III ABC-02 trial. Interestingly, weighted median of mOS increased from 9.7 to 11.7 months.

Health-related quality of life is a major concern in this palliative setting. Gemox regimen prescribed on day 1 every 2 weeks limits the number of visits compared to the standard regimen of cisplatin (25–35 mg/m^2^ administered on days 1 and 8) as in the pivotal phase III ABC-02 trial. However, the benefit of limited number of visits in terms of quality of life was not demonstrated. Moreover, this analysis indicated that cisplatin-based regimen was associated with a higher incidence of side effects in terms of asthenia, diarrhea, hepatotoxicity, and hematology. Nevertheless, oxaliplatin-based regimen was associated with more peripheral neuropathy which may have more significant detriment on patient quality of life than hematologic toxicity. Furthermore, while there was increased grade 3/4 neutropenia in patient treated with Gem/CDDP group, there was no difference in febrile neutropenia between the regimens. Therefore, the impact of these two regimens on quality of life cannot be clearly analyzed in our study and a longitudinal health-related quality of life analysis is necessary in a prospective randomized trial comparing these two regimens.

Our analysis has some limitations. First, a meta-analysis has not been conducted because these two regimens were never directly compared, so a direct statistical comparison was not feasible. Then, the methodological definitions of primary and secondary outcomes were unspecified in numerous studies. Among the 18 studies assessing Gem/CDDP, 16 trials analyzed the OS but only seven provided criteria defining OS. Among the three studies which analyzed PFS only two studies defined clearly PFS and two trials used the term “time-to-progression” with events of interest “death” and “progression” which is usually the definition of PFS (Table S2). Among the 15 studies assessing Gemox, 14 trials analyzed the OS but only eight studies provided criteria defining OS. Among the nine studies which analyzed PFS only five studies defined clearly PFS and one trial used the term “progression-free survival” with events of interest only progression which is usually the definition of time-to-progression (Table S3). Then, there is an the imbalance in stage of disease between the two groups with more patients at metastatic setting in the Gem/CDDP group than in the Gemox group, which may impact on toxicity especially hepatotoxicity and asthenia.

Finally, these results suggest that the Gem/CDDP regimen with cisplatin (25–35 mg/m^2^) administered on days 1 and 8 is associated with a short survival advantage than Gemox regimen (11.7 vs. 9.5 months). Our results should be interpreted cautiously and a further confirmatory prospective randomized trial between these two arms taking into account the impact of treatment on health-related quality of life is warranted.

## Supporting Information

**Table S1.** Adverse events (grade 3 and 4) according to treatment groups in the sensitivity analysis (including the six studies with cisplatin low dose [25–35 mg/m^2^] administered on days 1 and 8).

**Table S2.** Time-to-event definition in 18 studies assessing cisplatin/gemcitabine. **Table S3.** Time-to-event definition in 15 studies assessing oxaliplatin/gemcitabine.

## References

[1] Alvaro D, Bragazzi MC, Benedetti A, Fabris L, Fava G, Invernizzi P (2011). Cholangiocarcinoma in Italy: a national survey on clinical characteristics, diagnostic modalities and treatment. Results from the “Cholangiocarcinoma” Committee of the Italian Association for the Study of Liver disease. Dis. Liver Dis.

[2] Glimelius B, Hoffman K, Sjödén PO, Jacobsson G, Sellström H, Enander LK (1996). Chemotherapy improves survival and quality of life in advanced pancreatic and biliary cancer. Ann. Oncol.

[3] Rao S, Cunningham D, Hawkins RE, Hill ME, Smith D, Daniel F (2005). Phase III study of 5FU, etoposide and leucovorin (FELV) compared to epirubicin, cisplatin and 5FU (ECF) in previously untreated patients with advanced biliary cancer. Br. J. Cancer.

[4] Kornek GV, Schuell B, Laengle F, Gruenberger T, Penz M, Karall K (2004). Mitomycin C in combination with capecitabine or biweekly high-dose gemcitabine in patients with advanced biliary tract cancer: a randomised phase II trial. Ann. Oncol.

[5] Ducreux M, Van Cutsem E, Van Laethem JL, Gress TM, Jeziorski K, Rougier P (2005). A randomised phase II trial of weekly high-dose 5-fluorouracil with and without folinic acid and cisplatin in patients with advanced biliary tract carcinoma: results of the 40955 EORTC trial. Eur. J. Cancer.

[6] Androulakis N, Aravantinos G, Syrigos K, Polyzos A, Ziras N, Tselepatiotis E (2006). Oxaliplatin as first-line treatment in inoperable biliary tract carcinoma: a multicenter phase II study. Oncology.

[7] Eckel F, Schmid RM (2007). Chemotherapy in advanced biliary tract carcinoma: a pooled analysis of clinical trials. Br. J. Cancer.

[8] Valle J, Wasan H, Palmer DH, Cunningham D, Anthoney A, Maraveyas A (2010). Cisplatin plus gemcitabine versus gemcitabine for biliary tract cancer. N. Engl. J. Med.

[9] Sharma A, Dwary AD, Mohanti BK, Deo SV, Pal S, Sreenivas V (2010). Best supportive care compared with chemotherapy for unresectable gall bladder cancer: a randomized controlled study. J. Clin. Oncol.

[10] Lee J, Park SH, Chang H-M, Kim JS, Choi HJ, Lee MA (2012). Gemcitabine and oxaliplatin with or without erlotinib in advanced biliary-tract cancer: a multicentre, open-label, randomised, phase 3 study. Lancet Oncol.

[11] Malka D, Fartoux L, Rousseau V, Trarbach T, Boucher E, Fouchardiere CDL (2012). Gemcitabine and oxaliplatin (GEMOX) alone or in combination with cetuximab as first-line treatment for advanced biliary cancer: final analysis of a randomized phase II trial (BINGO). J. Clin. Oncol.

[12] Carraro S (2001). Gemcitabine and cisplatin in locally advanced or metastatic gallbladder and bile duct adenocarcinomas. Proc. Am. Soc. Clin. Oncol.

[13] Malik IA, Aziz Z, Zaidi SHM, Sethuraman G (2003). Gemcitabine and Cisplatin is a highly effective combination chemotherapy in patients with advanced cancer of the gallbladder. Am. J. Clin. Oncol.

[14] Baluch S, Lau J, dhilon T, Wasan HS (2003). A well tolerated and highly effective regimen for locally advanced and metastatic biliary tract cancers with gemcitabine and cisplatin. Proc. Am. Soc. Clin. Oncol.

[15] Reyes-Vidal J (2003). Gemcitabine (G) and cisplatin (C) in the treatment of patients (pts) with unresectable or metastatic gallbladder cancer: results of the phase II GOCCHI study 2000-13. Proc. Am. Soc. Clin. Oncol.

[16] Doval DC, Sekhon JS, Gupta SK, Fuloria J, Shukla VK, Gupta S (2004). A phase II study of gemcitabine and cisplatin in chemotherapy-naive, unresectable gall bladder cancer. Br. J. Cancer.

[17] Thongprasert S, Napapan S, Charoentum C, Moonprakan S (2005). Phase II study of gemcitabine and cisplatin as first-line chemotherapy in inoperable biliary tract carcinoma. Ann. Oncol.

[18] Kim ST, Park JO, Lee J, Lee KT, Lee JK, Choi S-H (2006). A Phase II study of gemcitabine and cisplatin in advanced biliary tract cancer. Cancer.

[19] Giuliani F, Gebbia V, Maiello E, Borsellino N, Bajardi E, Colucci G (2006). Gemcitabine and cisplatin for inoperable and/or metastatic biliary tree carcinomas: a multicenter phase II study of the Gruppo Oncologico dell'Italia Meridionale (GOIM). Ann. Oncol.

[20] Park BK, Kim YJ, Park JY, Bang S, Park SW, Chung JB (2006). Phase II study of gemcitabine and cisplatin in advanced biliary tract cancer. J. Gastroenterol. Hepatol.

[21] Lee J, Kim T-Y, Lee MA, Ahn MJ, Kim H-K, Lim HY (2008). Phase II trial of gemcitabine combined with cisplatin in patients with inoperable biliary tract carcinomas. Cancer Chemother. Pharmacol.

[22] Meyerhardt JA, Zhu AX, Stuart K, Ryan DP, Blaszkowsky L, Lehman N (2008). Phase-II study of gemcitabine and cisplatin in patients with metastatic biliary and gallbladder cancer. Dig. Dis. Sci.

[23] Charoentum C, Thongprasert S, Chewaskulyong B, Munprakan S (2007). Experience with gemcitabine and cisplatin in the therapy of inoperable and metastatic cholangiocarcinoma. World J. Gastroenterol.

[24] Valle JW, Wasan H, Johnson P, Jones E, Dixon L, Swindell R (2009). Gemcitabine alone or in combination with cisplatin in patients with advanced or metastatic cholangiocarcinomas or other biliary tract tumours: a multicentre randomised phase II study - The UK ABC-01 Study. Br. J. Cancer.

[25] Goldstein D, Gainford MC, Brown C, Tebbutt N, Ackland SP, van Hazel G (2011). Fixed-dose-rate gemcitabine combined with cisplatin in patients with inoperable biliary tract carcinomas. Cancer Chemother. Pharmacol.

[26] Okusaka T, Nakachi K, Fukutomi A, Mizuno N, Ohkawa S, Funakoshi A (2010). Gemcitabine alone or in combination with cisplatin in patients with biliary tract cancer: a comparative multicentre study in Japan. Br. J. Cancer.

[27] Weatherly J, Eckmann K, Patel D, Landgraf AN, Slade JH, Wolff RA (2011). Chemotherapy outcomes for the treatment of unresectable intrahepatic and hilar cholangiocarcinoma: a retrospective analysis. J. Clin. Oncol.

[28] Gebbia N, Verderame F, Di leo R, Santangelo D, Cicero G, Valerio M (2005). A phase II study of Oxaliplatin (O) and Gemcitabine (G) first line chemotherapy in patients with advanced biliary tract cancers. J. Clin. Oncol.

[29] Harder J, Riecken B, Kummer O, Lohrmann C, Otto F, Usadel H (2006). Outpatient chemotherapy with gemcitabine and oxaliplatin in patients with biliary tract cancer. Br. J. Cancer.

[30] Verderame F, Russo A, Di Leo R, Badalamenti G, Santangelo D, Cicero G (2006). Gemcitabine and oxaliplatin combination chemotherapy in advanced biliary tract cancers. Ann. Oncol.

[31] Manzione L, Romano R, Germano D (2007). Chemotherapy with gemcitabine and oxaliplatin in patients with advanced biliary tract cancer: a single-institution experience. Oncology.

[32] Kim HJ, Lee NS, Lee S-C, Bae SB, Kim CK, Cheon YG (2009). A phase II study of gemcitabine in combination with oxaliplatin as first-line chemotherapy in patients with inoperable biliary tract cancer. Cancer Chemother. Pharmacol.

[33] André T, Reyes-Vidal JM, Fartoux L, Ross P, Leslie M, Rosmorduc O (2008). Gemcitabine and oxaliplatin in advanced biliary tract carcinoma: a phase II study. Br. J. Cancer.

[34] Cassier PA, Thevenet C, Souquet J, Ponchon T, Baulieux J, Partensky C (2008). Outcome of patients receiving chemotherapy for advanced biliary tract or gallbladder cancer. J. Clin. Oncol.

[35] Jang J-S, Lim HY, Hwang IG, Song HS, Yoo N, Yoon S (2010). Gemcitabine and oxaliplatin in patients with unresectable biliary cancer including gall bladder cancer: a Korean Cancer Study Group phase II trial. Cancer Chemother. Pharmacol.

[36] Hollebecque A, Bouché O, Romano O, Scaglia E, Cattan S, Zerbib P (2010). Experience of gemcitabine plus oxaliplatin chemotherapy in patients with advanced biliary tract carcinoma. Chemotherapy.

[37] Sharma A, Mohanti B, Raina V, Shukla N, Pal S, Dwary A (2010). A phase II study of gemcitabine and oxaliplatin (Oxigem) in unresectable gall bladder cancer. Cancer Chemother. Pharmacol.

[38] Fiteni F, Nguyen T, Borg C, Dobi E, Fein F, Thierry-Vuillemin A (2011). Biliary tract carcinomas: a retrospective analysis of first line chemotherapy based on platinum compounds and second line based on 5 fluorouracil. Eur. Soc. Med. Oncol.

[39] Borad M, Reddy S, Bahary N, Uronis H, Sigal DS, Cohn AL (2012). Gastrointestinal tumors, non-colorectal. Ann. Oncol.

[40] Malka D, Fartoux L, Rousseau V, Trarbach T, Boucher E, Fouchardiere CDL (2012). Gemcitabine and oxaliplatin (GEMOX) alone or in combination with cetuximab as first-line treatment for advanced biliary cancer: final analysis of a randomized phase II trial (BINGO). J. Clin. Oncol.

