# Supplementary material for: Cisplatin/gemcitabine or oxaliplatin/gemcitabine in the treatment of advanced biliary tract cancer: a systematic review
Source: Cancer Med. 2014 Aug 11;3(6):1502–11. doi: 10.1002/cam4.299 (PMC4298376; doi:10.1002/cam4.299)
Supplement: Supplementary file 1 — Table S1. Adverse events (grade 3 and 4) according to treatment groups in the sensitivity analysis (including the six studies with cisplatin low dose [25–35 mg/m2] administered on days 1 and 8). [file cam40003-1502-sd1.doc]

**Supplementary Table 1.** Adverse events (grade 3 and 4) according to treatment groups in the sensitivity analysis (including the six studies with cisplatin low- dose (25-35 mg/m2) administered on days 1 and 8)

|  | **Cisplatine/ Gemcitabine (N=414)** | | | **Oxaliplatine/gemcitabine (N=699)** | | | **p** |
| --- | --- | --- | --- | --- | --- | --- | --- |
|  | **No. of missing studies** | **No. of available data** | **N (%)** | **No. of missing studies** | **No. of available data** | **N (%)** |
| **Asthenia** | 1 | 370 | 60 (16) | 8 | 408 | 23 (6) | <0.0001 |
| **Nausea** | 1 | 370 | 17 (5) | 10 | 331 | 12 (4) | 0.65 |
| **Vomiting** | 1 | 370 | 21 (6) | 6 | 519 | 18 (3) | 0.14 |
| **Diarrhea** | 2 | 166 | 13 (8) | 8 | 373 | 8 (2) | 0.004 |
| **Peripheral neuropathy** | 4 | 75 | 1 (1) | 3 | 543 | 58 (11) | 0.002 |
| **Alopecia** | 4 | 266 | 2 (0.8) | 13 | 97 | 0 (0) | 0.99 |
| **Renal toxicity** | 3 | 279 | 4 (1) | 13 | 97 | 0 (0) | 0.58 |
| **Hepatotoxicity** | 3 | 279 | 43 (15) | 9 | 360 | 24 (7) | 0.0006 |
| **Anemia** | 0 | 414 | 53 (13) | 8 | 300 | 17 (6) | 0.002 |
| **Thrombopenia** | 0 | 414 | 58 (14) | 6 | 473 | 34 (7) | 0.043 |
| **Neutropenia** | 0 | 414 | 119 (29) | 7 | 442 | 52 (12) | <0.0001 |
| **Mucositis** | 4 | 92 | 1 (1) | 11 | 241 | 0 (0) | 0.28 |
| **Febrile neutropenia** | 3 | 125 | 4 (3) | 10 | 331 | 13 (4) | 0.83 |
