# Supplementary material for: Cisplatin/gemcitabine or oxaliplatin/gemcitabine in the treatment of advanced biliary tract cancer: a systematic review
Source: Cancer Med. 2014 Aug 11;3(6):1502–11. doi: 10.1002/cam4.299 (PMC4298376; doi:10.1002/cam4.299)
Supplement: Supplementary file 2 [file cam40003-1502-sd2.doc]

**Supplementary Table 2:** Time-to-event definition in 18 studies assessing Cisplatin/gemcitabine

| **Study** | **Primary endpoint** | **Time-to-event endpoint assessed** | **Definitions of time-to-event endpoints** | | | | | | | | | | |
| --- | --- | --- | --- | --- | --- | --- | --- | --- | --- | --- | --- | --- | --- |
|  |  |  | **Overall Survival** | | | **Progression-free survival** | | | | **Time to progression** | | | |
|  |  |  | **Starting point** | | **Event** | **Starting point** | | **Event** | | **Starting point** | | **Event** | |
|  |  |  | **Randomisation** | **First treatment** | **Death** | **Randomisation** | **First treatment** | **Death** | **Progression** | **Randomisation** | **First treatment** | **Death** | **Progression** |
|  |  |  |  |  |  |  |  |  |  |  |  |  |  |
| Carraro et al., 2001 | NA | OS, TTP | - | - | - | - | - | - | - | - | - | - | - |
| Malik et al., 2003 | NA | OS, TTP | - | - | - | - | - | - | - | - | - | - | - |
| Baluch et al., 2003 | NA | no time to event endpoint assessed | - | - | - | - | - | - | - | - | - | - | - |
| Reyes Vidal et al., 2003 | response rate | OS | - | - | - | - | - | - | - | - | - | - | - |
| Doval et al., 2004 | response rate | OS, TTP | - | X | X | - | - | - | - | - | X | X | X |
| Thongprasert et al., 2005 | NA | OS, TTP, duration of response | - | X | X | - | - | - | - | - | X | X | X |
| Kim et al., 2006 | response rate | OS, TTP | - | X | X | - | - | - | - | - | X | - | X |
| Giuliani et al., 2006 | response rate | OS, TTP | - | X | X | - | - | - | - | - | X | - | X |
| Park et al., 2006 | NA | OS, TTP, duration of response | - | - | - | - | - | - | - | - | - | - | - |
| Lee et al., 2006 | NA | OS, TTP | - | X | X | - | - | - | - | - | X | - | X |
| Meyerhardt A. et al., 2007 | response rate | OS, PFS | X | - | X | X | - | X | X | - | - | - | - |
| Chaorentum et al., 2007 | response rate | OS | - | - | - | - | - | - | - | - | - | - | - |
| Lee et al., 2008 | response rate | OS, TTP | - | - | - | - | - | - | - | - | - | - | - |
| Valle et al., 2009 | 6-month progression-free rate | no time to event endpoint assessed | - | - | - | - | - | - | - | - | - | - | - |
| Valle et al., 2010 | OS | OS, PFS | X | - | X | X | - | X | X | - | - | - | - |
| Goldstein et al., 2010 | response rate | OS, TTP | - | - | - | - | - | - | - | - | - | - | - |
| Okusaka et al., 2010 | 1-year survival rate | OS, PFS | - | - | - | - | - | - | - | - | - | - | - |
| Weatherly et al., 2011 | response rate | OS | - | - | - | - | - | - | - | - | - | - | - |

**Supplementary Table 3:** Time-to-event definition in 15 studies assessing oxaliplatin/gemcitabine

| **Study** | **Primary endpoint** | **Time-to-event endpoint assessed** | **time-to-event endpoint** | | | | | | | | | | |
| --- | --- | --- | --- | --- | --- | --- | --- | --- | --- | --- | --- | --- | --- |
|  |  |  | **OS** | | | **PFS** | | | | **TTP** | | | |
|  |  |  | **Starting point** | | **Event** | **Starting point** | | **Event** | | **Starting point** | | **Event** | |
|  |  |  | **Randomisation** | **First treatment** | **Death** | **Randomisation** | **First treatment** | **Death** | **Progression** | **Randomisation** | **First treatment** | **Death** | **Progression** |
|  |  |  |  |  |  |  |  |  |  |  |  |  |  |
| Gebbia et al.,2005 | NA | OS, TTP | - | - | - | - | - | - | - | - | - | - | - |
| Verderame et al.,2006 | response rate | OS | - | - | - | - | - | - | - | - | - | - | - |
| Harder et al., 2006 | response rate | OS, TTP | - | X | X | - | - | - | - | - | X | - | X |
| Manzione et al., 2007 | NA | OS, TTP | - | - | - | - | - | - | - | - | - | - | - |
| Cassier et al., 2008 | NA | PFS | - | - | - | - | - | - | - | - | - | - | - |
| Kim et al., 2008 | response rate | OS, TTP | - | X | X | - | - | - | - | X | - | - | X |
| Andre et al., 2008 | response rate | OS, PFS | - | X | X | - | X | X | X | - | - | - | - |
| Sharma et al.,. 2009 | OS, response rate, toxicity | OS, PFS | X | - | X | - | - | - | - | X | - | - | X |
| Sharma et al., 2010 | OS | OS, PFS | X | - | X | X | - | - | X | - | - | - | - |
| Jang et al., 2010 | response rate | OS, TTP | - | X | X | - | - | - | - | - | X | - | X |
| Hollebecque et al., 2010 | NA | OS, PFS | - | X | X | - | X | X | X | - | - | - | - |
| Fiteni et al., 2010 | OS | OS, PFS | - | X | X | - | X | X | X | - | -- | - | - |
| Lee et al., 2011 | PFS | OS, PFS | - | - | - | X | - | X | X | - | - | - | - |
| Phelip et al., 2012 | NA | OS, PFS | - | - | - | - | - | - | - | - | - | - | - |
| Malka et al., 2012 | 4-month progression-free rate | OS, PFS | - | - | - | - | - | - | - | - | - | - | - |
